# Progress in the Genetics of Myelodysplastic Syndromes with a Latin American Perspective

**DOI:** 10.3390/genes16060687

**Published:** 2025-06-02

**Authors:** Ana Lisa Basquiera, Verónica Andreoli, Sofía Grille, Carolina Bárbara Belli

**Affiliations:** 1 Hospital Privado Universitario de Córdoba, Instituto Universitario de Ciencias Biomédicas de Córdoba (IUCBC), X5016 Córdoba, Argentina; veronica.andreoli@hospitalprivadosa.com.ar; 2 Unidad Académica de Hematología, Hospital de Clínicas, Universidad de la República, 11300 Montevideo, Uruguay; sgrille@hc.edu.uy; 3 Instituto de Medicina Experimental (IMEX-CONICET/ANM), C1425 AUM Buenos Aires, Argentina; cbelli@hematologia.anm.edu.ar

**Keywords:** myelodysplastic syndrome, NGS, mutations, prognostic, germline

## Abstract

Myelodysplastic syndromes (MDS) are a heterogeneous group of hematological malignancies characterized by ineffective hematopoiesis, resulting in cytopenias, morphologic dysplasia in hematopoietic lineages, and a variable risk of progression to acute myeloid leukemia. Significant advances in the understanding of MDS have been made in recent years, largely due to the implementation of molecular tools. Latin America is a highly diverse region, both ethnically and racially, and often faces resource limitations that challenge the broad applicability of recent advances in MDS. In this review, we discuss the key genes implicated in the pathogenesis and classification of MDS, and their relevance to diagnosis, prognosis, and potential therapeutic targets. We also explore the challenges associated with the identification of germline predisposition to MDS in Latin America and discuss the current availability and limitations of molecular diagnostic tools in the region.

## 1. Introduction

Myelodysplastic syndromes (MDS) are a heterogeneous group of hematological malignancies characterized by ineffective hematopoiesis, leading to cytopenia, morphologic dysplasia in the hematopoietic lineages, and a variable progression rate to acute myeloid leukemia (AML) [1]. Clinical manifestations are related to cytopenia (infections due to leukopenia, bleeding due to thrombocytopenia, and/or symptoms related to anemia), and with AML in cases of transformation. For all patients with MDS, the median overall survival (OS) is approximately 5 years, but it can range from less than 1 year to 10 years, depending on the specific biological characteristics and the presence of significant comorbidities [2,3]. The neoplastic nature of the disease is highlighted by its rate of progression to AML, which is, in fact, the leading cause of death in these patients [4]. Despite the development of drugs in the field, allogeneic hematopoietic stem cell transplantation remains the only curative treatment [5].

MDS are composed of multiple neoplastic clones arising from hematopoietic stem and progenitor cells [6]. Based on this concept, the most recent World Health Organization (WHO) classification renamed MDS as myelodysplastic neoplasms, while retaining the traditional abbreviation [7]. Classifications in MDS have evolved following the advances in cytogenetic and molecular findings, with implications for diagnosis, prognosis, and therapeutic algorithms [1,7,8]. As MDS are associated with aging, they predominantly affect the elderly, and the global burden of MDS appears to be increasing, particularly in Latin America [9].

The impact of race and ethnicity on MDS outcomes has been studied in the United States using data from the Surveillance, Epidemiology, and End Results (SEER) program, revealing significant differences in age at diagnosis, disease risk, and survival outcomes based on racial/ethnic backgrounds [10]. Non-Hispanic white patients were older (59.5% ≥75 years old) than Hispanics (46.4% ≥75 years old), and Hispanic patients presented more frequently with high-risk disease. Median OS was better in non-Hispanic Black patients compared to Hispanic and non-Hispanic White patients (33 vs. 28 vs. 25 months, respectively); an interaction between race/ethnicity and age groups was noted [10].

Latin America is a region comprising several countries, covering an area of approximately 19,197,000 square kilometers. As of 2024, the population of Latin America and the Caribbean reached 663 million people [11,12]. The population of Latin America arose from a genetic admixture of African, Native American, and European ancestries [13]. As a result, the Latin American genome is highly diverse and relatively underrepresented in molecular studies of MDS. Understanding the genetic alterations in MDS patients in this region is crucial to evaluating their impact on disease phenotype and prognosis. In the molecular setting, different polymorphisms in the nucleotide excision repair pathway were studied in 173 Brazilian and 96 Argentine patients with MDS [14]. That study reinforces the heterogeneity of MDS and highlights the importance of ethnic differences and regional influences in the pathogenesis and prognosis of MDS. Accordingly, it has been suggested that incorporating ethnic diversity, through the evaluation of genetic ancestry, into prognostic models is essential to ensure their universal applicability [15].

In the clinical context, the first multicenter study from Latin America, which included 1080 patients with MDS (median age 69 years, with 75% over the age of 60 at diagnosis), reported differences in clinical features and outcomes among countries [16]. Despite regional variability, the most widely used clinical prognostic scores for MDS were validated in Latin American patients [2,16,17], and the efficacy of specific treatments, such as erythropoietin, lenalidomide, hypomethylating agents (HMA), and allogeneic stem cell transplantation [18,19,20,21,22,23], was also confirmed.

We aimed to describe the advances in understanding the pathogenesis and molecular genetics of MDS that have led to the current classification for diagnosis and prognostic risk stratification models in MDS, focusing on the molecular landscape in Latin America. Given the significant academic activity related to MDS in Latin America [12], we will include the experience gained in this field when available, as well as the current knowledge gaps and region-specific challenges.

## 2. Overview of Molecular Characterization Studies

Globally, over the past 20 years, the use of next-generation sequencing (NGS) has significantly advanced the understanding of MDS [24]. The recognition of a high frequency of mutations in genes involved in RNA splicing [25] expanded the number of known affected pathways in MDS, which now include epigenetic regulation, signal transduction, DNA damage, transcription factors, and cohesin complexes, among others [26]. Also, mutations can be classified as early or late events in the progression of MDS [26,27]. At least one gene mutation can be found in over 90% of cases when analyzing a panel of approximately 50 recurrently mutated genes [28]. In agreement with the findings of Papaemmanuil et al., the series by Haferlach et al. identified 47 significantly mutated genes, with *TET2*, *SF3B1*, *ASXL1*, *SRSF2*, *DNMT3A*, and *RUNX1* mutated in over 10% of cases [27,28]. More recently, Bernard et al. studied molecular alterations in 2957 patients with MDS, profiling mutations in 152 genes [29]. This study included 110 Brazilian patients. Bernard et al. confirmed that at least one oncogenic genomic alteration was present in 94% of patients with MDS. A total of 3186 cytogenetic alterations were reported in 41% of patients, and 9254 oncogenic mutations across 121 genes were identified in 90% of patients [29].

In Latin America, targeted efforts have been made to gather data and describe the mutational burden of MDS in this population. The first multicenter study from Latin America included 145 patients with MDS and 37 patients with chronic myelomonocytic leukemia (CMML) (median age 65 years, male-to-female ratio 1:3) from Argentina and Uruguay [30]. This retrospective real-world study used six different commercially available myeloid-focused NGS-based panels, covering 30–63 genes per panel, with molecular data generated at each institution. The results showed that 81% of patients had at least one detectable variant, and that individuals with CMML exhibited a significantly higher mutational burden compared to those with MDS (3.5 variants vs. two variants, respectively). The five genes with the highest mutation frequency were *TET2*, *ASXL1*, *SRSF2*, *SF3B1*, and *DNMT3A* [30]. Similarly, a recent study conducted in Uruguay analyzed 52 patients with MDS or CMML (median age 67 years) and found that 75% harbored at least one somatic mutation. The most frequently mutated genes were *DNMT3A*, *TP53*, *TET2*, *ASXL1*, and *SF3B1*. Copy number variations (CNVs) were detected in 38% of cases, including abnormalities that were not identifiable by conventional cytogenetics. Patients with ≥3 mutations had higher bone marrow blast percentages (median, 7% vs. 1%, *p* = 0.005), elevated lactate dehydrogenase (LDH) levels (*p* = 0.0027), and increased International Prognostic Scoring System–Revised (IPSS-R) scores (*p* = 0.013) compared to those with fewer mutations. Moreover, this group demonstrated significantly reduced OS (*p* = 0.0032) and leukemia-free survival (LFS) (*p* = 0.001). These findings underscore the prognostic relevance of mutational burden in MDS and support its integration into contemporary risk stratification models [31].

## 3. Key Molecular Mechanisms Driving MDS Pathogenesis

**Figure 1:**
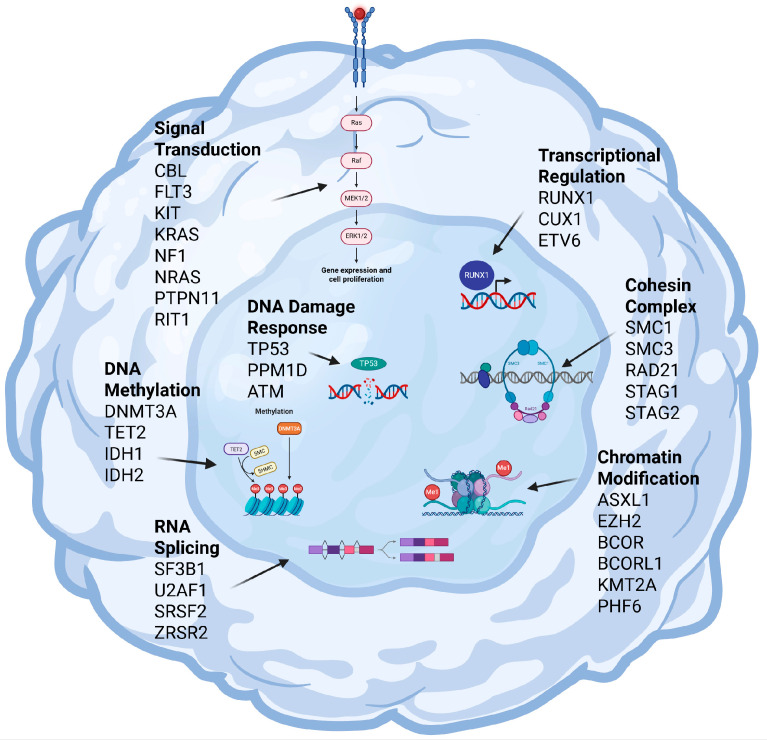
Schematic representation of a cell illustrating the genes implicated in the pathogenesis of myelodysplastic syndromes (MDS). Advances in high-throughput/next-generation sequencing (NGS) have uncovered a broad spectrum of mutations, expanding our understanding of disease pathogenesis. The genes depicted are categorized based on their involvement in crucial cellular pathways, including RNA splicing, chromatin modification, DNA methylation, transcriptional regulation, DNA damage response, signal transduction, and cohesin complexes. This diagram highlights the molecular heterogeneity of MDS, emphasizing the intricate interplay of genetic factors contributing to disease initiation and progression.

Molecular findings in MDS have contributed to characterizing the disease, understanding its pathogenesis, and the interactions between the involved genes. Figure 1 presents a schematic representation of the main genetic and epigenetic pathways implicated in MDS.

### 3.1. RNA Splicing

Splicing factor mutations are known to arise early in the development of MDS [27]. These proteins are essential for the assembly of major U-2 type spliceosomes or minor U-12 type spliceosomes, which catalyze the splicing of pre-mRNA. Mutations in these splicing factors can lead to aberrant alternative splicing events [32]. They have been observed in approximately 50% of the patients with MDS and are frequent in cases progressing to AML [25,33,34,35,36]. These mutations rarely co-occurred, with the most frequent involving splicing factor 3b subunit 1 (*SF3B1*), serine- and arginine-rich splicing factor 2 (*SRSF2*), U2 small nuclear RNA auxiliary factor 1 (*U2AF1*), and Zinc finger CCCH-type, RNA binding motif, and serine/arginine rich 2 (*ZRSR2*) [25]. Having multiple spliceosome mutations does not appear to worsen prognosis compared to having a single mutation [37].

Mutations in *SF3B1* in MDS lead to distinct splicing changes, defining a phenotype characterized by ring sideroblasts, ineffective erythropoiesis, a lower rate of leukemic transformation, and longer OS, in the absence of other unfavorable prognostic markers [29,38,39]. In a bioinformatic analysis of an RNA-seq data set (GSE114922) of CD34+ cells from patients with MDS and harboring *SF3B1* variants, Lincango et al. identified 1342 differentially expressed genes when compared with patients lacking other splicing factor mutations and healthy controls [40]. Functional enrichment analysis (gseGo and enrichGo) revealed dysregulated pathways involving ribosomes, oxidative phosphorylation, mitochondrial gene expression, and translation [40]. Among them, *NDUFA8*, *RBM25*, *MRRF*, and *AC*, related to mitochondrial pathways and telomeric maintenance, were confirmed to be significantly dysregulated. This novel gene Hub explained 77.9% of the variance associated with *SF3B1* mutations in Argentine MDS patients [41].

MDS-*SF3B1* is now recognized as a distinct entity, comprising more than 90% of MDS cases with ≥5% ring sideroblasts [7,8,39]. Lincango et al. reported *SF3B1* mutations in 16% of patients from Argentina and Uruguay [30], doubling the previously reported frequency in Brazil [42]. Catalán et al. found a 13% mutation rate among Uruguayan patients with MDS to be associated with older age (median age 76 years), elevated ferritin levels, and lower bone marrow blast counts compared to those without *SF3B1* mutations [31]. MDS can also occur alongside systemic mastocytosis. In such complex clinical scenarios, treatment is guided by the prevailing clinical features. Lenalidomide has shown efficacy in a patient with *SF3B1*-mutant MDS and systemic mastocytosis, achieving transfusion independence after HMA failure and before receiving a transplant, based on the complexity of the accompanying mutational profile [43].

Mutations in *U2AF1* are primarily found at two hotspots (S34F, Q157) within zinc finger domains [25]. Patients with the S34F mutations appear to have better survival than those with the Q157 variant, including those undergoing allogeneic stem cell transplantation [44]. *U2AF1* mutations have been associated with del(20q), chromosome 7 alterations, higher rates of transfusion-dependent anemia, and an excess of bone marrow blasts [45]. Typically, these mutations emerge later in life and are correlated with accelerated progression to MDS and AML [44].

Mutations in *SRSF2*, usually affecting the Pro95 residue and associated with aberrant exon-skipping alternative splicing patterns, were found in 12–17% of patients with MDS [28,34]. They are linked to advanced age, male sex, normal cytogenetics, and CMML subtype [25,34,46,47]. A South American series reported a 19% frequency, likely reflecting the inclusion of CMML patients [30]. Bersanelli et al. described two prognostic clusters enriched for *SRSF2* variants: one tied to *TET2*, and another to *ASXL1*, *RUNX1*, *IDH2*, or *EZH2*. Both are linked to trisomy 8 and monocytosis; however, the second cluster showed more cytopenias, multilineage dysplasia, and higher blast percentages, indicating a worse prognosis [45].

*ZRSR2* mutations are less frequent (2–10%) in MDS but are significantly increased in high-risk compared to low-risk cases [25,27]. Although their impact on AML progression and OS is less pronounced [48], *ZRSR2* mutations, as well as other splicing factors, are classified among MDS-related gene mutations by both the International Consensus Classification (ICC) and the WHO classification of AML [7,8].

### 3.2. Chromatin Modification

Mutations in the Additional Sex Combs Like 1 (*ASXL1*) gene occur in 20.7% of MDS cases, with approximately 70% being frameshift mutations and 30% heterozygous point mutations that result in translational alterations [49]. *ASXL1* mutations are frequent in clonal hematopoiesis and, therefore, recognized as early events in leukemogenesis, with increased dominance of mutant clones identified in secondary AML after MDS [50]. *ASXL1* mutations are indicative of poor prognostic outcomes in patients with MDS [51].

Enhancer of Zeste Homolog 2 (*EZH2*) variants in myeloid neoplasms correspond to loss-of-function mutations and have been reported in approximately 5% of patients with MDS [52]. Patients with MDS and *EZH2* mutations are typically older and show lower response rates to HMA compared to those with wild-type *EZH2*. Co-occurrence of *ASXL1* or *RUNX1* mutations in patients with *EZH2*-mutated MDS correlates with shorter OS in comparison with those without such co-mutations [53].

The homologous genes *BCOR* (BCL6 Corepressor) and *BCORL1* (BCL6 Corepressor-like 1) are situated on the X chromosome [54]. *BCOR* primarily acts by repressing myeloid regulatory genes while supporting lymphoid lineage development [55]. *BCOR* mutations are most frequently identified in patients between the sixth and seventh decades of life and are associated with low white blood cell counts at diagnosis. *BCOR* mutations are found in 3–8% of the patients with MDS, with *RUNX1* and *U2AF1* being among the most frequently co-mutated genes, while *TP53* mutations were less commonly observed. Mutations in the *BCOR* gene are distributed throughout the exon sequence, with no identifiable hotspot [56]. The presence of a complex karyotype was associated with reduced OS in cases with *BCOR*-mutated AML/MDS, whereas treatment with allogeneic hematopoietic transplantation has demonstrated improved survival [56]. The *BCORL1* mutations have been reported in approximately 1–5% of MDS cases and are more frequent in patients with prior therapy [57].

The human *KMT2A* gene, located on chromosome 11q23, is recurrently rearranged in acute leukemias [58]. In contrast, *KMT2A* partial tandem duplication (PTD) involves intragenic duplications and has been described in approximately 2% of patients with MDS, where it is associated with a poor response to HMA and an adverse outcome [59,60].

*PHF6* is thought to modulate gene expression by modifying chromatin, acting both as a cancer suppressor and influencing the differentiation of hematopoietic lineages. *PHF6* mutations have been reported in 0.7% to 5% of myeloid malignancies, including MDS [61]. A variant allele frequency (VAF) above 20% for *PHF6* mutations in patients with MDS has been significantly correlated with worse OS and disease progression [61,62].

The cohesin complex, comprising *SMC1*, *SMC3*, *RAD21*, and either *STAG1* or *STAG2*, is a recurrent mutational target, with *STAG2* (stromal antigen 2) mutations accounting for more than half of cohesin mutations in myeloid malignancies [63]. These mutations were mostly mutually exclusive and occurred in 10% of MDS cases [64]. *STAG2*, located on chromosome Xq25, is considered one of eight secondary-type mutation markers of secondary AML (including *SRSF2*, *SF3B1*, *U2AF1*, *ZRSR2*, *ASXL1*, *EZH2*, and *BCOR*) [36]. *STAG2* mutations are linked with high-risk disease and adverse outcomes in MDS, despite sensitivity to HMA. Katamesh et al. found that in 18% of MDS cases that progressed to AML, an increased co-mutational burden was present, which drives progression, suggesting that *STAG2* mutations promote leukemic transformation by inducing genetic instability and facilitating the acquisition of additional alterations [65]. Morphologically, a striking form of megakaryocytic dysplasia has been reported in a patient diagnosed with MDS with increased blasts and multiple pathogenic *STAG2* mutations [66].

### 3.3. DNA Methylation

DNA methyltransferase 3 A (*DNMT3A*) mutations are the most frequent alterations in de novo AML (20–25%), MDS (about 10%), and clonal hematopoiesis of indeterminate potential (CHIP), and are mostly heterozygous [67]. Reduced *DNMT3A* function results in global genomic hypomethylation, particularly enriched at promoters near the transcription start sites of genes involved in cellular differentiation [68,69]. The most commonly variants in *DNMT3A*, along with *TET2* and *ASXL1* mutations, are key drivers of age-related clonal hematopoiesis [70]. *DNMT3A* variants are reported in 17% of patients with MDS and are enriched in *SF3B1*-mutated cases, particularly in women [71,72]. The *DNMT3A* R882 mutation is a known hotspot, and MDS patients harboring this mutation exhibit more severe leukopenia, frequent co-mutations in *SRSF2* and *IDH2*, a higher frequency of excess blasts, a markedly increased rate of progression to AML, and inferior progression-free-survival (PFS) compared to non-R882 mutant MDS cases [73].

Ten-eleven translocation (TET) family proteins, particularly *TET1*, *TET2*, and *TET3*, can change DNA by oxidizing 5-methylcytosine (5mC). *TET2*, specifically, is a key regulator of DNA methylation that is primarily responsible for converting 5-methylcytosine to 5-hydroxymethylcytosine during DNA demethylation [74]. The loss or attenuation of TET function is implicated in genomic hypermethylation and transcriptional reprogramming that promotes oncogenesis [75]. Smith et al. reported 71 *TET2* mutations in 12% of 355 patients with MDS. Mutant clones were present in T cells, as well as CD34(+) and total bone marrow cells. The presence of *TET2* mutations was not significantly associated with WHO subtypes, IPSS score, cytogenetic abnormalities, or AML transformation in terms of prognosis [76]. However, biallelic *TET2* mutations may be associated with adverse outcomes [77] and may drive monocytic differentiation, consistent with their prevalence in CMML [78].

Isocitrate dehydrogenase 1 or 2 (*IDH1* or *IDH2*) mutations are detected in approximately 12% of patients with MDS and are enriched in high-risk cases and severe neutropenia [79,80]. These recurrent mutations affecting critical metabolic enzymes result in the production of the oncometabolite 2-hydroxyglutarate (2-HG), which promotes leukemogenesis through a block in normal myeloid differentiation [80]. Mutation frequencies vary by MDS morphological subtype: 4% in refractory anemia with ring sideroblasts, 12% in refractory cytopenia with multilineage dysplasia, 14% in MDS-unclassifiable, 14% in refractory anemia with excess blasts RAEB-1, and 23% in RAEB-2. *IDH1* mutations were linked with inferior survival, whereas *IDH2* mutations did not affect survival [81,82].

### 3.4. Transcriptional Regulation

The transcription factor *RUNX1* is mutated in familial platelet disorder with associated myeloid malignancy, as well as in sporadic MDS and leukemia [83,84]. Approximately 10% of patients with MDS harbor *RUNX1* mutations, which are related to AML transformation [6,85] and poor OS [24,86]. In patients with low-risk MDS, *RUNX1* mutations are associated with disease progression, even in the absence of overt AML transformation [83]. *RUNX1* expression levels may further refine prognostication, with higher *RUNX1* expression associated with shorter LFS and OS [87]. *RUNX1* has also been implicated in autoinflammatory features observed in patients with MDS [88,89].

The CUT-like homeobox 1 (*CUX1*) gene, mapped to chr7q22.1, encodes multiple CUX1 isoforms, one of which is the full-length p200 *CUX1* protein [90]. A broad range of genes and microRNAs involved in various cellular functions are regulated by *CUX1* transcription factors [91]. In addition, tumor suppression by *CUX1* partly occurs through the regulation of genes controlling the cell cycle and DNA repair [90,91]. Most patients with MDS and AML with −7/del(7q), and up to 15% of MDS patients and 5% of AML patients diploid for the *CUX1* locus, showed decreased *CUX1* expression. In 75% of cases with *CUX1* mutations, the mutations were heterozygous, while microdeletions, homozygous mutations, and compound-heterozygous mutations were observed less frequently. *CUX1* mutations or deletions were correlated with inferior survival compared with *CUX1* wild-type [92]. A high association with dysplastic changes has been reported [90].

The *ETV6* gene (previously named *TEL*) belongs to the ETS (E26 transformation-specific) family of transcription factors [93]. Although rare, *ETV6* mutations are recurrent somatic events in myeloid neoplasms and are associated with a poor prognosis in MDS [24]. Point mutations in the *ETV6* gene have been identified in 2.7% of patients with MDS, were frequently subclonal, and never occurred in isolation, suggesting a role as a late event in disease progression [24,94]. Co-occurrence of *ASXL1*, *SETBP1*, *RUNX1*, and *U2AF1* mutations was frequently observed in *ETV6*-mutated cases, compared to those with wild-type *ETV6* [94].

### 3.5. DNA Damage Response

*TP53*, recognized as the “guardian of the genome”, is a tumor suppressor gene. This gene is the most commonly altered in human cancer [95]. In MDS, *TP53* mutations are identified in 12% of cases [29]. Patients with multiple *TP53* mutations or with both mutation and loss of heterozygosity (LOH), either due to chromosomal deletion or copy-neutral LOH (cnLOH) as a result of an acquired uniparental disomy, are classified as *TP53* multi-hit mutations. The multi-hit state (biallelic inactivation) represents two-thirds of MDS cases with TP53 mutations, and 91% of these are associated with a complex karyotype [96]. Alterations affecting *TP53* can be detected by conventional cytogenetics, typically by evidencing loss of chromosome 17 or its short arm (17p), including deletions or unbalanced translocations involving the *TP53* locus 17p13.3. Other techniques, such as fluorescence in situ hybridization (FISH) or chromosomal microarrays, can identify deletions and, depending on the design of the microarray, cnLOH. Targeted NGS detects mutations and provides the VAF, with VAFs greater than 50% suggesting additional alterations in the remaining locus [97]. *TP53* multi-hit events are among the strongest predictors of adverse outcomes [29] and are recognized as a distinct entity in the WHO 5th edition classification [7]. Similarly, the ICC defines *TP53*-mutated MDS based on the presence of two mutations with a VAF > 10%, or one mutation associated with a complex karyotype (typically involving 17p-), VAF > 50%, or cnLOH, in patients with bone marrow blasts below 10%. For those with higher blast percentages, one alteration with VAF > 10% is sufficient for classification [8].

In a recently published South American series, 18 (10%) of 182 patients with MDS harbored *TP53* mutations, and five (27.7%) of these 18 were classified as bi-*TP53* based on either the presence of two mutations or a VAF > 50%. Limited access to advanced complementary technologies may hinder the detection of other types of alterations [30]. Another retrospective study focused specifically on *TP53*-mutated MDS patients from Latin America reported a mean age of 68 years, with 65% having de novo MDS. In addition, 58% of the patients were categorized as high or very high risk based on the IPSS-R, with a median OS of 16 months. The mean VAF of *TP53* was 33%, with 59% of patients harboring multi-hit mutations and 30% showing a complex karyotype [98]. In Uruguay, Catalán et al. reported a *TP53* mutation prevalence of 21.2% in MDS, higher than the typically reported prevalence at diagnosis. Notably, 70% of patients with *TP53*-mutated MDS exhibited multi-hit alterations, accounting for 14.9% of the overall cohort. These patients displayed significantly worse clinical features, including lower hemoglobin levels, increased transfusion dependence, elevated ferritin levels, thrombocytopenia, and had significantly poorer OS and LFS [31].

*PPM1D* is a phosphatase that plays a central role in the DNA repair response as a component of a regulatory feedback loop with *TP53*. Activated *TP53* induces *PPM1D* expression, which then both directly and indirectly dephosphorylates *TP53*, leading to downregulation of *TP53*-mediated apoptosis. Mutations in *PPM1D*, often consisting of nonsense or frameshift variants in exon 6, result in a gain-of-function that constitutively inhibits *TP53* activation in response to DNA damage. The increased and stabilized expression of these truncated variants provides a survival advantage to hematopoietic clones by rendering them resistant to DNA-damaging agents, such as cisplatin or the topoisomerase inhibitor etoposide [99]. *PPM1D* mutations are commonly observed in therapy-related clonal hematopoiesis, as well as therapy-related AML and MDS. These mutations may arise in the founding clone, with 81% expanding under exposure to alkylating agents [100]. In the aforementioned South American series, only a minority of patients were tested for *PPM1D* mutation, as this gene was only recently incorporated into clinical diagnostic panels [30].

### 3.6. Signal Transduction

The genes *CBL*, *FLT3*, *KIT*, *KRAS*, *NF1*, *NRAS*, *PTPN11*, and *RIT1* are key components of cellular signaling pathways in MDS [26]. Somatic mutations involved in signaling seem to be a prerequisite for the transformation of MDS to AML, particularly *NRAS/KRAS*, *FLT3*, *CBL*, and *PTPN11* [101,102]. In a cohort of 72 patients with MDS, 15 (20.8%) tested positive for *FLT3* internal tandem duplication (FLT3-ITD) and showed significantly inferior OS and PFS compared to those without the mutation [103]. Single-gene mutations in the *RAS* pathway are relatively rare in MDS, and their clinical significance remains unclear. Both *NRAS* and *KRAS* have been linked with disease progression in this neoplasm [104]. Ren et al. studied 370 patients with MDS and identified *RAS* pathway mutations in 57 (15.4%) of them. Patients with *RAS* mutations had a higher median percentage of bone marrow blasts, a greater number of co-mutated genes, were more frequently categorized as higher-risk according to IPSS-R, and showed a higher AML transformation rate compared to those with MDS and wild-type *RAS*. Although *RAS* mutations did not significantly affect the response to disease-modifying treatments, OS was significantly shorter in patients harboring these mutations compared to those with wild-type *RAS* [105].

### 3.7. Other Mechanisms

One of the hallmarks of MDS is aberrant DNA hypermethylation, particularly affecting genes involved in DNA repair. In a cohort of Brazilian patients with MDS, the Ataxia-telangiectasia mutated (*ATM*) gene exhibited higher methylation levels among individuals who progressed to AML. Furthermore, *ATM* expression progressively declined from low-risk MDS subtypes to high-risk MDS and AML. These findings indicate that *ATM* is often silenced or downregulated in MDS, likely as a result of promoter hypermethylation or gene mutations [106].

Among the enzymes involved in histone modification and gene expression, histone deacetylases have a critical role [107]. Within this group, the Sirtuins (*SIRTs*) family of enzymes has shown diverse functions in transcriptional regulation, metabolic control, and genome maintenance [108]. Goes et al., in a study of 80 bone marrow samples from Brazilian patients with MDS, reported altered *SIRT* expression patterns associated with specific clinical and prognostic features of MDS [109].

## 4. Molecular Genetics Data Integrated for Prognostic Risk Stratification in MDS, Including Artificial Intelligence

Molecular information in MDS has been integrated into a comprehensive model combining clinical parameters and cytogenetics findings: the IPSS-R for Molecular data (IPSS-M) [29] based on the earlier IPSS-R [110]. This model, developed by the International Working Group for prognosis in MDS, includes molecular information on 31 genes and categorizes patients into six risk categories with distinct prognostic implications. The IPSS-M outperformed the IPSS-R and allowed for the reclassification of 46% of patients [29]. Nevertheless, emerging data on specific entities, such as MDS with *DDX41* mutations, suggest that the IPSS-M may not be applicable in all cases [111].

Artificial intelligence is playing an increasing part in the prognostication of hematological malignancies, including MDS [112]. The Spanish Group of Myelodysplastic Syndromes developed an Artificial Intelligence Prognostic Scoring System for MDS (AIPSS-MDS), using data from 7202 patients. The best model was built on eight traditional variables: age, gender, hemoglobin, leukocyte count, platelet count, neutrophil percentage, bone marrow blast percentage, and cytogenetic risk group. This model accurately predicted OS and PFS and showed superiority over both the IPSS-R and the age-adjusted IPSS-R [113].

Data from Latin America are based on Lincango et al., who compared the performance of the IPSS-M with various other prognostic systems, including molecular prognostic scores such as the European MDS (EuroMDS) clustering system and the Munich Leukemia Laboratory (MLL) models, as well as non-molecular prognostic scores, such as the AIPSS-MDS and IPSS-R [29,30,45,110,113,114]. This retrospective study included 182 patients (>18 years old) with a diagnosis of myelodysplastic neoplasms between 2009 and 2022 at five centers in Argentina and one in Uruguay [30]. The IPSS-M model predicted OS more accurately than other molecular-based models [30].

Interestingly, the AIPSS-MDS performed similarly to IPSS-M, with slight differences depending on whether the prognostic scores were analyzed as continuous or grouped into risk categories [30]. AIPSS-MDS showed greater prognostic accuracy than the IPSS-R. When simplifying classification into low- and high-risk groups, 51% of patients categorized as low-risk (<3.5) by the IPSS-R would have been reclassified as high-risk according to the AIPSS-MDS, with no cases being downstaged. In contrast, only 13% of patients were upstaged from low-risk by IPSS-R to high-risk by IPSS-M, while 5% were downstaged and might have been considered for lower-intensity treatment. Using the median risk score from the original training set, AIPSS-MDS appears to better identify low-risk patients with longer OS [30,113]. This model may represent a more suitable prognostic tool than the IPSS-R, particularly in the resource-limited settings in our region, where molecular testing is not routinely available due to infrastructure and reimbursement constraints [30].

The increased use of NGS and more sophisticated molecular techniques, with or without the application of machine learning approaches, has enabled the development of new prognostic models based exclusively on genetics, and, consequently, a deeper understanding of MDS pathogenesis and the complex networks of gene interactions [114,115,116]. Kewan et al., using a combination of NGS and unsupervised machine learning, described a personalized risk evaluation that was independent of disease duration and stage, and the blast count [115]. The finding of molecular clusters has enabled the recognition of cases with convergent molecular mechanisms, delineating a rationale for appropriate treatments [115]. A comparison between full genetics-based and machine-learning-based models is shown in Table 1.

## 5. Molecular Data Incorporated in Current Diagnostic Classifications and New Proposals

Significant progress in the understanding and diagnosis of myeloid neoplasms is introduced in the fifth edition of the WHO classification of hematolymphoid tumors [7]. Particularly in the setting of MDS, the WHO classification now recognizes newly defined entities such as clonal hematopoiesis and broadly divides MDS into two categories (1) MDS with defining genetic abnormalities and (2) MDS defined morphologically. The category of MDS with defining genetic abnormalities includes the following entities: MDS with biallelic *TP53* inactivation, MDS with low blasts and *SF3B1* mutation (MDS-*SF3B1*), and MDS with low blasts and del(5q) [117]. In turn, the ICC of myeloid neoplasms also highlights *TP53* and *SF3B1* mutations, establishing them as distinct entities [8]. The latter classification also retains MDS-related molecular findings for patients with bone marrow blasts between 10% and 19%, as described by Lindsley et al., who harbor mutations in splicing factors, *STAG2*, *EZH2*, *ASXL1*, *BCOR*, and additionally *RUNX1* [8,36]. In the remaining cases classified under the ICC, diagnostic categorization is based on blast counts [8].

In this regard, mutational profile, gene associations, and co-mutation patterns may lead to the identification of even more new entities (Table 1). Since blast count remains as a key parameter distinguishing MDS and AML, while softened in the presence of AML-related abnormalities, it is a subjective measure, and the threshold between low and elevated blasts can be controversial. Huber et al. identified nine genetically defined, mutually exclusive hierarchical subgroups in MDS: (1) biallelic *TP53* mutations (bi*TP53*), (2) complex karyotype, (3) mutated *RUNX1*, (4) mutated *ASXL1*, (5) deletion of 5q [del(5q)], (6) mutated *SF3B1*, (7) mutations in *U2AF1*, *SRSF2*, and/or *ZRSR2* (splicing factor-positive, SP+), (8) presence of at least one mutation in *DNMT3A*, *TET2*, or other genes recurrently mutated in MDS (splicing factor-negative with ≥1 mutation, SP−/≥1), and (9) complete absence of any of the above genetic markers (splicing factor-negative without mutations, SP−/0) [114].

Kewan et al. identified 14 distinct molecular clusters by applying an unsupervised machine learning method to 3588 patients with MDS and secondary AML [115]. Patients harboring the same mutation or cytogenetic alteration may be assigned to different molecular clusters—each with a distinctive molecular signature—depending on the co-occurrence of additional genomic abnormalities. Their model performed independently of clinical and morphological features and demonstrated clinical relevance in terms of OS (five risk categories), regardless of IPSS-M [115].

**Figure 2:**
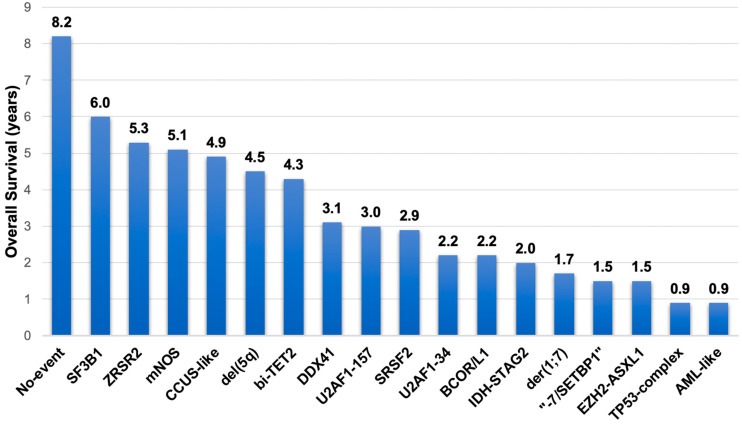
Overall survival according to a novel molecular taxonomy proposed by Bernard et al. [116]. mNOS = Molecularly not otherwise specified; CCUS = clonal cytopenia of undetermined significance; bi-*TET2* = biallelic *TET2*; AML = acute myeloid leukemia.

More recently, Bernard et al. explored the role of genetic alterations in defining distinct disease subtypes using 3233 representative MDS patient samples (median age, 72 years) from the IPSS-M cohort and a 152-gene targeted NGS panel integrated with cytogenetic analysis [116]. Genetically defined diagnostic subtypes (MDS-5q, MDS-*SF3B1*, or MDS-bi*TP53*) accounted for 23% and 22% of cases according to the WHO 2022 and ICC classifications, respectively. Molecular genetic analysis revealed 10,564 oncogenic mutations in 126 different genes (in 91% of patients), 3558 chromosomal alterations (in 43% of patients), and 375 cnLOH events (in 11% of patients). Sixteen molecular subgroups, comprising 86% of the patient population, and two phenotypically distinct residual groups were identified through the analysis (Table 2), further refining the molecular taxonomy of the MDS [116,118]. Figure 2 shows the corresponding median OS for each group.

## 6. Molecular Data to Tailor Treatment Strategies

Despite the emergence of new therapies, no significant improvement in outcomes has been observed in Latin America over the past two to three decades [4,119]. Data from the U.S. National Cancer Database showed that OS has not improved over time, except in younger patients (<40 years old) [120]. These findings may not yet reflect the impact of molecular profiling on treatment decisions. Indeed, molecular data provide not only prognostic information but are also essential for guiding targeted therapies. For example, *SF3B1*-positive variants respond to luspartercept in the low-risk MDS [121]. *IDH1*-mutation-positive patients may benefit from ivosidenib [122] and *IDH2*-mutation-positive patients from enasidenib [123]. *TP53* mutations may serve as markers of a lack of response to lenalidomide and predictors of relapse after transplantation [5]. Somatic mutations in splicing factor genes, *NPM1*, *IDH1*, and *IDH2*, are also likely predictive of response to venetoclax [124,125,126]. Additionally, patients carrying *DDX41* mutations have shown exceptional responses to venetoclax combined with HMA [127]. Although data remain limited and sometimes conflicting, *TET2* and/or *DNMT3A* mutation and the *ASXL1* wild-type status were predictors of better responses to HMA [128,129,130]. When deciding which patients should undergo transplantation, the IPSS-M prognostic index has proven to be the most accurate tool [131]. Patients with a low risk and those with moderate-low risk benefited from delaying transplantation, while in those with higher risk, immediate transplantation improved survival [131]. Current algorithms incorporate molecular data to guide transplant timing and predict post-transplant outcomes in transplant-eligible patients [132].

## 7. Germline Predisposition: Implications and Challenges in Latin America

Recent progress in genomic research has highlighted that a significant proportion of patients diagnosed with MDS carry germline pathogenic variants in genes linked to susceptibility to hematologic malignancies. This discovery has markedly shifted the traditional paradigm, as MDS was previously considered solely an acquired disorder. Today, it is estimated that between 4% and 10% of MDS cases are linked to germline mutations, with higher frequencies observed in younger individuals and those with a family history indicative of inherited susceptibility. In pediatric populations, familial or inherited forms of AML and MDS may account for up to 50% of cases, while in young adults, the figure ranges from 10% to 20%, decreasing to approximately 5–10% in older adults [133,134,135,136,137,138]. Furthermore, in a cohort of 404 patients with MDS undergoing allogeneic stem cell transplantation, at least one pathogenic germline variant was detected in 7% of cases, with the highest detection rate among adolescents [139]. Importantly, these numbers may underestimate the true prevalence, as many studies are limited by incomplete gene panels, lack of evaluation for copy number or noncoding variants, and exclusion of patients without confirmed germline status.

**Figure 3:**
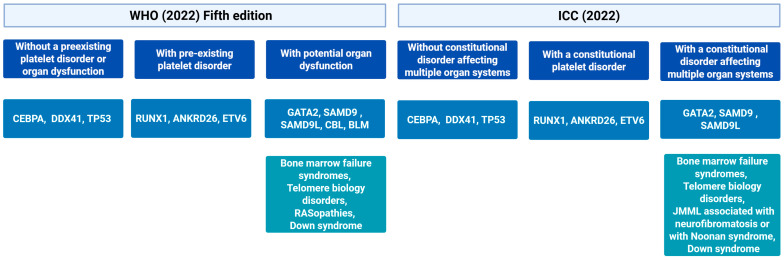
Classification of Myeloid Neoplasms Associated with Germline Predisposition, comparing the World Health Organization (WHO) 5th edition (2022) and the International Consensus Classification (ICC) (2022). The diagram categorizes myeloid neoplasms based on the presence of germline predisposition, distinguishing between conditions without preexisting platelet disorders or organ dysfunction, those associated with constitutional platelet disorders, and those linked to multi-organ systemic disorders. The genetic variants implicated in each classification are listed accordingly. JMML: Juvenile Myelomonocytic Leukemia.

The clinical and molecular landscape of germline predisposition to myeloid malignancies is highly heterogeneous. These conditions may result from variants inherited from a parent or arise de novo during gametogenesis or early embryonic development. Modes of inheritance vary depending on the gene involved, and include autosomal dominant, autosomal recessive, and X-linked patterns [140]. The penetrance, age at onset, and spectrum of hematologic or systemic manifestations differ markedly between syndromes. The significance of these germline syndromes was acknowledged with the addition of a dedicated chapter in the 2016 revision of the WHO classification of hematopoietic neoplasms [141], reflecting growing clinical awareness and increased availability of NGS. The WHO 2022 classification and the ICC further expanded the list of recognized syndromes [7,8]. Figure 3 shows the 2022 WHO classification and the ICC of myeloid neoplasms with germline predisposition.

### 7.1. Main Syndromes

Given the overlap between sporadic and inherited cases in terms of clinical presentation, comprehensive molecular profiling and detailed family history are essential for identifying germline cases (Table S1). Timely recognition of these conditions has clinical implications for clinical management, including treatment, transplant donor selection optimization, and also genetic familial counseling and cancer surveillance in at-risk relatives. As research progresses, refining the criteria for germline testing and integrating them into diagnostic pathways will be crucial for improving outcomes of patients with MDS and other myeloid neoplasms.

Familial AML associated with germline *CEBPA* mutations is one of the most widely recognized syndromes. These patients typically develop leukemia in early to middle adulthood, often without preceding hematologic abnormalities [142,143,144]. Although the precise prevalence is unknown, familial cases may represent up to 1% of all AML, particularly in the context of a normal karyotype [145]. *CEBPA* mutations usually affect the N-terminal domain and exhibit an autosomal dominant pattern of inheritance with high penetrance. A second-hit mutation in the opposite allele is commonly required for leukemogenesis [142,143]. These leukemias generally respond well to standard therapy, but careful donor selection is essential, as using related donors with the same germline variant has resulted in donor-derived leukemia [144].

Another well-characterized syndrome is associated with germline mutations in *DDX41*, which encodes a DEAD-box helicase involved in RNA processing. Studies have found *DDX41* mutations in 1.5–2.5% of adults with MDS or AML, making it one of the most common hereditary myeloid neoplasia syndromes [146]. This condition predominantly affects older adults and is often asymptomatic until the onset of MDS or AML. Although the penetrance appears high, many affected individuals lack a family history of hematologic malignancies, likely due to age-related expression [147]. Germline mutations of *DDX41*, located at 5q35.3, most commonly include missense and frameshift variants [146], although large deletions have also been reported [148]. Most cases involve an inherited truncating variant with a second somatic hit in the opposite allele. Interestingly, cytogenetic analysis has shown that the acquisition of a del(5q) may represent the second hit in these patients [149]. Ongoing clinical trials are evaluating the potential benefits of monitoring unaffected *DDX41* mutation carriers, focusing on detecting low-frequency secondary somatic mutations in *DDX41* [150]. Treatment of hematologic malignancies associated with germline *DDX41* variants is generally similar to that of other forms of AML and MDS [146]. Importantly, these patients may develop more severe graft-versus-host disease after transplantation, particularly when receiving grafts from wild-type donors [151].

Germline mutations in *RUNX1* cause familial platelet disorder with a predisposition to myeloid malignancy. With an autosomal dominant inheritance, it is characterized by lifelong mild-to-moderate thrombocytopenia and impaired platelet function, often accompanied by an increased risk of developing MDS, AML, or T-acute lymphoblastic leukemia (T-ALL) [152,153]. Affected individuals carry a markedly increased lifetime risk of hematologic malignancies, ranging from 35–40%, with wide variability in age at diagnosis. The phenotypes of MDS, AML, and T-ALL in familial platelet disorder vary widely [152,154,155,156,157]. Leukemic transformation commonly involves a second somatic hit in *RUNX1* or cooperating mutations (e.g., in *GATA2*), and cytogenetic abnormalities such as del(7q), 3q aberrations, or gain of an additional chromosome 21 bearing the mutations [149].

Other hereditary syndromes involve genes such as *ANKRD26* and *ETV6*, both presenting with autosomal dominant thrombocytopenia and an increased predisposition to myeloid malignancies, including MDS and acute leukemia (AL). Patients typically present with lifelong mild to moderate thrombocytopenia and normal platelet size, often without significant bleeding complications. Despite the usually mild hemorrhagic phenotype, the underlying clonal instability confers a substantial long-term risk of malignant transformation [158]. *ANKRD26*-related thrombocytopenia (THC2) is an uncommon condition, with fewer than 100 pedigrees described based on available literature. Pathogenic variants typically occur in the 5′ untranslated region (5′UTR) of the gene, causing abnormal upregulation of *ANKRD26* in megakaryocytes, defective platelet production, and a cumulative risk of MDS or AML estimated at 8–20% [159,160,161]. Germline *ETV6* mutations are similarly rare, with an estimated prevalence of less than 1% among individuals with inherited thrombocytopenia. They may also be associated with a broader cancer predisposition, including lymphoid malignancies and, less frequently, solid tumors. The long-term risk of hematologic malignancy in *ETV6* mutation carriers ranges from 30% to 40%, though penetrance and age of onset are highly variable [162,163,164].

*GATA2* deficiency syndrome presents with a heterogeneous clinical spectrum and may be linked to MDS, AL, bone marrow failure, congenital neutropenia, or other hematologic abnormalities. It is one of the more prevalent inherited disorders associated with AL and MDS, particularly in children and young adults. *GATA2* deficiency encompasses a wide range of clinical phenotypes, including immunodeficiency, lymphedema, and myeloid neoplasia [165,166]. Many patients develop MDS or AML by their third decade, often presenting with monosomy 7/del(7q), trisomy 8, or other cytogenetic abnormalities such as der(1;7)(q10;p10), although 35% of cases show a normal karyotype [149,167]. Germline mutations in *GATA2*, located at chromosome band 3q21.3, may include missense, nonsense, or truncating frameshift mutations, large genomic rearrangements, or alterations within a conserved enhancer element in intron 5 [168]. Commercial panels not designed to detect intronic sequences or whole-exome approaches may miss these alterations. Surveillance and timely transplantation are critical in these patients due to progressive cytopenia and the risk of severe infections.

*SAMD9* and *SAMD9L* germline mutations are major contributors to pediatric MDS, especially in the presence of monosomy 7 and inherited bone marrow failure, where it is detected in up to 20% of patients [169,170]. These gain-of-function mutations inhibit hematopoiesis, occasionally resulting in the spontaneous loss of the affected chromosome, which may restore hematopoietic function but also increases the risk of MDS. Clinical presentations can include MIRAGE syndrome (acronym for *m*yelodysplasia, *i*nfection, *r*estriction of growth, *a*drenal hypoplasia, *g*enital phenotypes, and *e*nteropathy) and ataxia-pancytopenia phenotypes. Management of these patients requires a multidisciplinary approach due to the complex clinical manifestations [171].

Although *TP53* mutations are more frequently associated with solid tumors, germline alterations in this gene also confer a rare but significant predisposition to hematologic malignancies, especially in individuals with Li-Fraumeni syndrome. Characterized by an autosomal dominant inheritance, this disorder is commonly associated with early-onset breast cancer, sarcomas, brain neoplasms, and adrenocortical carcinomas; however, MDS and AL can occur in a subset of carriers without prior solid tumors, with a median age of first cancer diagnosis between 20 and 30 years [172]. It has been suggested that young individuals with biallelic *TP53* mutations should be tested to rule out a germline variant [173]. A germline founder variant of *TP53* p.R337H has been described in patients from southern Brazil, with a higher frequency than other *TP53* mutations [174,175]. The identification of these variants in specific populations allows for the design of screening and long-term follow-up, given the lifelong risk that carriers have of developing Li–Fraumeni syndrome [176,177]. Interestingly, clinical manifestations in carriers of this mutation have been diverse, and an indirect modulatory effect of microRNA (miRNA) on the *TP53* expression has been proposed [178].

### 7.2. When to Suspect and How to Diagnose a Germline Predisposition

Genetic testing is becoming increasingly essential in the diagnostic workup of patients with AML or MDS when germline predisposition is suspected. While no single global consensus exists, published guidelines from the Nordic MDS Group and the National Comprehensive Cancer Network (NCCN) provide key clinical criteria to guide testing [5,179]. The Spanish group has also published guidelines online, covering broad aspects from laboratory testing to genetic counseling and clinical management [180]. These guidelines emphasize the importance of germline assessment of patients diagnosed with MDS/AML before the age of 50, particularly when there is a personal or family history indicative of an inherited predisposition. According to the recommendations of the Nordic group, diagnostic testing is advised for patients with: (1) two first- or second-degree relatives with MDS/AML or chronic thrombocytopenia, with at least one diagnosed before age 50; (2) a proband with MDS/AML and two relatives with solid tumors, one of them under 50; or (3) a diagnosis before age 50 associated with features of bone marrow failure syndromes or cytogenetic alterations, particularly involving chromosome 7 [179]. The NCCN guidelines reinforce these and expand the indications to include: (1) monosomy 7 or other chromosome 7 abnormalities in patients under 50; (2) hypocellular MDS or aplastic bone marrow at diagnosis; (3) a clinical suspicion of predisposition at any age; and (4) family donors in the context of allogeneic transplantation or candidates for transplantation with clinical signs of inherited bone marrow failure syndromes [5]. Both guidelines also recommend confirmatory germline testing from non-hematopoietic tissues when somatic mutations with a VAF suggestive of germline origin (typically >40%) are detected. Germline testing is particularly essential when selecting related donors for hematopoietic stem cell transplantation to avoid the risk of donor-derived leukemia [5,179].

Genetic testing should ideally be coordinated by clinicians or genetic counselors with expertise in hereditary cancer syndromes and the interpretation of genomic data [181]. Testing strategies can be either targeted or broad. Broader approaches, such as whole-genome sequencing (WGS) or whole-exome sequencing (WES), may be particularly useful when no familial diagnosis has been established or when clinical features suggest multiple possible conditions. Targeted testing through gene panels is appropriate when a specific mutation has been identified in a relative or when molecular features strongly suggest a known syndrome. Depending on the method, single-nucleotide variants (SNVs) as well as small insertions and deletions, called indels, can be identified. To identify larger genomic rearrangements and CNV, complementary techniques such as microarrays, multiplex ligation-dependent probe amplification (MLPA), or optical genome mapping (OGM) may be necessary. Gene selection and methodology vary by laboratory, underscoring the importance of choosing appropriate strategies based on clinical context [182]. In urgent scenarios, such as a pending stem cell transplant, testing may need to be expedited or done in parallel.

In patients with hematologic malignancy, the preferred specimen for germline testing is typically cultured skin fibroblasts, as blood or bone marrow may be contaminated by malignant or clonal hematopoietic cells. Hair follicles are also recommended, particularly by the Spanish guidelines [180]. Saliva, buccal swabs, and nails may be used in some cases, but have limitations due to DNA yield or potential contamination. Blood may be used in remission or from patients not previously transplanted, but clonal hematopoiesis must be considered [5,179,183].

In Latin America, despite limited resources and infrastructure, early efforts are underway to better characterize germline predisposition in MDS, though data remain limited. Most Latin American countries face significant barriers to routine germline testing, including limited availability of NGS panels validated for constitutional DNA, high costs, and a shortage of expertise in variant interpretation. Consequently, underdiagnosis of hereditary syndromes is likely. However, some centers, such as Uruguay, Brazil, and Argentina, have begun to implement protocols for analyzing germline tissues and are developing targeted panels or applying WES to detect germline alterations. Notably, collaborations between the University of Chicago and Hospital das Clínicas (São Paulo, Brazil), on *ANKRD26* and *ETV6* [184], and the development of a machine learning algorithm that helps with the diagnosis of bone marrow failure in collaboration with centers from Brazil, with the National Institute of Health (NIH) [185] have been established. Further collaborations include studies on *RUNX1* between Argentina and Italy [186] and participation in international consortia on familial platelet disorder with associated myeloid malignancy [187]. Reports from Brazil and Argentina have described individual cases or small series involving germline mutations in *RUNX1*, *GATA2*, *DDX41*, and telomere biology genes. In Uruguay, additional reports described complex hereditary backgrounds, such as dual germline mutations in *RUNX1* and *DDX41* [188], and familial cases with *CEBPA* mutations affecting donor selection for transplantation [189,190]. These examples underscore the feasibility and clinical relevance of integrating germline analysis even in resource-limited settings.

A survey conducted by Grupo Latinoamericano de Mielodisplasia (GLAM) across 21 centers in 12 countries revealed that only 28% of laboratories (6/21) had the capacity to confirm the germline origin of variants [191], mainly due to a lack of access to confirmatory testing using non-hematopoietic tissues (e.g., fibroblast culture). This gap has serious implications, particularly for genetic counseling, familial risk assessment, and transplant donor selection. In a region where related donors are frequently used, failure to detect germline mutations can negatively impact outcomes.

To address this gap, it is essential to establish regional or national reference centers, define standardized criteria for germline testing, and promote clinician and laboratory training in variant interpretation. Strengthening referral networks and fostering collaborative frameworks will ensure timely and accurate diagnosis. Initiatives such as GLAM play a key role in raising awareness, building technical capacity, and promoting equitable access to molecular diagnostics across the region.

## 8. Molecular Tools in Latin America

Challenges in diagnosing and treating MDS persist, largely due to significant economic and technological disparities across and within Latin American countries [12,192]. In 2015, the GLAM was established as a multidisciplinary group of healthcare professionals dedicated to the study of MDS, working in close collaboration with regional hematology societies [12]. In alignment with its goals, GLAM conducted an initial survey involving 458 respondents from nine Latin American countries (Argentina, Bolivia, Chile, Colombia, Dominican Republic, Ecuador, Paraguay, Peru, and Uruguay). The results revealed heterogeneous access to the conventional cytogenetic analysis (53% to 100%) and flow cytometry (42% to 100%), despite efforts toward standardized implementation [12,192,193]. Subsequently, the GLAM MDS registry, known as Re-GLAM, showed that NGS panels were used in only 15.2% of the registered patients [194]. To evaluate the real-world application of laboratory practices concerning NGS technology, a 33-question survey was distributed and responses collected between March 2022 and January 2023. The survey addressed various aspects of NGS implementation across Latin American institutions. In total, 37 centers in 12 countries were contacted, with 21 out of 26 providing nearly complete responses.

**Figure 4:**
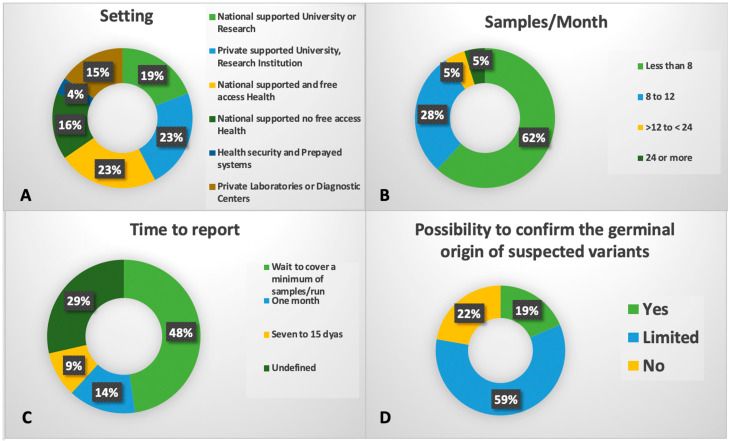
Data from a survey conducted by GLAM in Latin America to learn about laboratory practices regarding NGS technology. (**A**) Type of institution performing NGS studies. (**B**) Number of samples per month processed by each laboratory. (**C**) Time to report NGS results. (**D**) Possibility of confirming germline variants following somatic NGS panel results.

The responding centers were fairly evenly distributed between private and publicly funded institutions, including universities, health or research centers, and clinical laboratories (Figure 4). Among them, 11 centers focused on adult patients, while 8 had mixed-age patient populations. Initial funding sources included governmental support (n = 12), the American Society of Hematology (ASH) (n = 2), private investment (n = 11), and combined funding mechanisms (n = 5), including International Consortium on Acute Leukemia (ICAL) projects (n = 4). Most laboratories reported using Illumina platforms (73%) and commercial NGS panels (69%), employing either amplicon- or capture-based technologies, primarily developed by Illumina (n = 9) or Sophia Genetics (n = 7). In terms of expertise, 9 out of 21 centers reported having less than one year of experience or being in the early stages of NGS implementation. The majority (12 out of 21; 57%) of centers adapted multiple guidelines to report their findings, although six lacked a clearly defined set of guidelines. Other results are shown in Figure 4. We consider this effort a foundational step toward optimizing laboratory capacities, promoting the exchange of best practices, and facilitating the transfer of scientific and technical expertise across Latin America [191].

## 9. Conclusions and Perspectives

In the era of a molecular revolution with the widespread use of NGS technology, comprehensive analyses of the genetic heterogeneity in MDS have guided advances in the diagnostic workup over the last 15 years. These improvements have enabled the recognition of disease subgroups linked to disease phenotypes, along with prognostic information that influences clinical treatment decisions. Genetically defined entities, such as MDS with del(5q), MDS with *SF3B1* mutation, and the important role of *TP53* inactivation, have been included in both the WHO 2022 classification and the ICC. Both systems have removed the ≥20% blast threshold in the presence of genetic abnormalities that define AML and, in turn, incorporated the concept of clonal hematopoiesis. The recognition of the importance of germline predisposition gives insights for outlining disease models and their therapeutic management.

Risk stratification and prognostic evaluation are essential for the effective management of patients who are commonly assessed using the IPSS-R. Mutational information is also being incorporated into prognostic scoring systems, with IPSS-M as a key tool for personalized risk assessment and guiding treatment decisions. Among other molecular-based proposals for classification, the recently developed molecular taxonomy of MDS is expected to enhance the implementation of precision medicine approaches.

The main challenge related to these latest advances is to provide access to MDS patients for genomic profiling. In the past few years, new laboratories have adopted the NGS technology, and collaborative projects are underway. However, only a minority of our patients get access to this, which clearly indicates that the needs of Latin American patients with MDS are unmet. Ideally, access to new technologies would extend across all of Latin America and not remain confined to specialized centers. As emphasized by Dr Cazola and Dr Malcovati [195], achieving this goal will involve the development of specialized infrastructures within healthcare systems, supported by close collaboration among healthcare providers, academia, and the life sciences industry across Latin America.

## Figures and Tables

**Table 1:** Comparative data between novel classification and/or prognostic stratification studies.

Author/Year	N/Diagnosis	Region	Methods	Median Follow-Up	Main Findings
Kewan et al./2023 [115]	3588/MDS and sAML	USA and Europe	NGS (40 genes) + ML	NI	MDS classification, prognostication, and prediction of treatment response, based solely on genetic factors (based on karyotype and mutation status). Fourteen molecularly distinct clusters. Correlation with OS (independent of IPSS-M) and response to treatment.
Huber et al./2023 [114]	735/De novo MDS	Europe (Germany)	WGS	9.3 years	MDS classification and prognostication based solely on genetic. Nine genetically defined, mutually exclusive hierarchical subgroups (based on karyotype and mutation status). Correlation with OS and IPSS-M risk groups.
Mosquera et al./2023 [113]	7202/MDS and sAML	Spain	ML	4.9 years	Enhanced MDS prognostication based on non-molecular variables (traditional clinical and laboratory). The model, AIPSS-MDS, performed better than IPSS-R and similar to IPSS-M. Correlation with OS and LFS.
Bernard et al./2024 [116]	3233/MDS or related disorders	USA, Europe, and Japan	NGS (152-gene panel)	NI	Classification and prognostication based on gene mutations, copy-number alterations, and copy neutral loss of heterozygocity. Sixteen molecular groups and two residual groups with negative findings, with different OS. Prognosis of t-MDS and myelodysplastic/myeloproliferative neoplasms depended on genetic subtypes.
Lincango et al./2024 [41]	182/MDS or related disorders	Latin America (AR and UY)	NGS (various gene panels)	1.9 years	AIPSS-MDS validation for prognosis, showing similar prognostic discrimination to IPSS-M. AIPSS-MDS useful in resource-limited centers without molecular testing.

Abbreviations: MDS = myelodysplastic syndrome; NGS = next generation sequencing; WGS = whole genome sequencing; ML = machine learning; OS = overall survival; sAML = secondary acute myeloid leukemia; IPSS-M = molecular international prognostic system; NI = not informed; IPSS-R = revised international prognostic system; LFS = leukemia-free survival; AIPSS-MDS = artificial intelligence prognostic scoring system for myelodysplastic syndrome; t-MDS = therapy-related myelodysplastic syndrome; AR = Argentine; UY = Uruguay.

**Table 2:** Molecular subgroups of myelodysplastic syndromes according to Bernard et al. [116] *.

Group	Entity	%	Reported Features
Validated five established subgroups	*DDX41*	3.3	56% of patients with mutated *DDX4*1 had both a putative germline *DDX41* variant (defined here as >30% VAF) and a somatic *DDX41* mutation, 37% had only a putative germline *DDX41* variant, and 7% had only somatic *DDX41* mutations.
	AML-like	2.0	*NPM1* mutations or at least 2 events from *WT1*, *FLT3*, *MLL*^PTD^, or *MYC* mutations.
	*TP53* complex	10.0	Multi-hit *TP53* mutations were present in 74% of cases, of which 91% had a complex karyotype.
	del(5q)	6.9	Presence of del(5q) as the sole cytogenetic abnormality or with 1 additional abnormality excluding −7/7q. Monoallelic *TP53* mutations were significantly enriched in this group.
	*SF3B1*	14.0	Indolent clinical course.
Confirmed three previously reported subgroups	Bi-allelic *TET2*	13.0	Early biallelic *TET2* mutations with splicing factor mutations in 80% of patients, most commonly affecting *SRSF2*, *SF3B1*, or *ZRSR2*. Modulation of phenotype by *ASXL1* and *RAS* mutations driving monocytosis and *JAK2* driving thrombocytosis.
	der(1;7)	0.5	*ETNK1* mutations were enriched in this group.
	CCUS-like	6.9	46% had a single mutated gene (*TET2* or *DNMT3A*), 8% had loss of Y without gene mutations, and 6% had only ≥2 DTA mutations.
Eight novel subgroups	“−7/*SETBP1*”	4.9	*SETBP1* mutations and/or −7 in the absence of complex karyotype. *GATA2* variants were prevalent.
	*EZH2-ASXL1*	4.0	*ASXL1* and *EZH2* mutation co-occurrence. High molecular complexity (75% of patients with ≥5 mutated genes).
	*IDH-STAG2*	8.9	Mutations at the *IDH2* R140 hot spot, *IDH1*, and/or *STAG2* co-occurring with either *SRSF2* or *ASXL1* mutations
	*BCOR/L1*	3.5	83% of patients had mutations in *BCOR*, 33% in *BCORL1*, and 17% in both genes.
	*U2AF1*	4.3	58% had a Q157 mutation, 41% had a S34 mutation, and 1% had both.
	*SRSF2*	2.2	Aggressive disease.
	*ZRSR2*	1.3	Indolent clinical course.
Two subgroups without defining genetic events	Not otherwise specified	7.9	Presence of other cytogenetic abnormalities and/or mutations in 51 other recurrently mutated genes.
	No event	6.5	Absence of any recurrent drivers evaluated.

* Abbreviations: AML: acute myeloid leukemia; CCUS: clonal cytopenia of undetermined significance; DTA: *DNMT3A/TET2/ASXL1*.

## Supplementary Materials

The following supporting information can be downloaded at: https://www.mdpi.com/article/10.3390/genes16060687/s1, Table S1: Hereditary Syndromes Associated with Myeloid Malignancies.

## Abbreviations

The following abbreviations are used in this manuscript:

MDS	Myelodysplastic syndromes
AML	Acute myeloid leukemia
AL	Acute leukemia
WHO	World Health Organization
ICC	International Consensus Classification
SEER	Surveillance, Epidemiology, and End Results
CMML	Chronic myelomonocytic leukemia
NGS	Next-generation sequencing
CNV	Copy number variations
IPSS-R	International Prognostic Scoring System Revised
IPSS-M	International Prognostic Scoring System Molecular
OS	Overall Survival
LFS	Leukemia Free Survival
VAF	Variant allele frequency
HMA	Hypomethylating agents
CHIP	Clonal hematopoiesis of indeterminate potential
PFS	Progression free survival
AIPSS	Artificial Intelligence Prognostic Scoring System
