# Supplementary material for: Progress in the Genetics of Myelodysplastic Syndromes with a Latin American Perspective
Source: Genes (Basel). 2025 Jun 2;16(6):687. doi: 10.3390/genes16060687 (PMC12192143; doi:10.3390/genes16060687)
Supplement: Supplementary file 1 [file genes-16-00687-s001.zip › genes-3622349-supplementary.pdf]

Table S1: Hereditary Syndromes Associated with Myeloid Malignancies [111,133-136,181].

Gene or Disorder	Disorder Name	Prevalence	Malignancy Type	Characteristic Clinical Manifestations
CEBPA	Familial AML with mutated *CEBPA*	~1% of AML	AML	No systemic manifestations
DDX41	Familial MDS/AML with mutated *DDX41*	Common among familial MDS/AML in older adults	MDS, AML, CMML	Cytopenias, male predominance, late onset
TP53	Li-Fraumeni syndrome	1 in 5000–20000	AML, MDS, ALL, many solid tumors	Early-onset cancers, breast, brain, sarcomas
MBD4	Familial AML with *MBD4* mutations	Rare	AML	Colorectal polyps, uveal melanoma, possible cancer predisposition
RUNX1	Familial platelet disorder with predisposition to myeloid malignancy	~1.5-4.5% of AML/MDS	MDS, AML, ALL	Thrombocytopenia, platelet dysfunction
ANKRD26	Thrombocytopenia 2	Rare 1/250 AML	AML, MDS, CML, MPN, ALL	Thrombocytopenia, pale platelets, platelet dysfunction leukocytosis
ETV6	Thrombocytopenia 5	Rare	ALL, MDS, AML, MM	Thrombocytopenia, macrocytosis, bleeding tendency
GATA2	*GATA2* deficiency syndrome	Rare. 7% of primary pediatric MDS	MDS, AML, CMML	Immunodeficiency, lymphedema, deafness, cytopenias.
SAMD9/SAMD9L	MIRAGE / Ataxia-pancytopenia syndromes	Rare. 20% of pediatric cases of BM failure/ MDS with monosomy 7	MDS, AML	Organ hypoplasia, neurologic symptoms, MIRAGE syndrome, monosomy 7.
SRP72	Familial aplastic anemia/MDS	Rare	MDS, aplastic anemia	Hearing loss
MECOM/EVI1	*MECOM*-associated syndrome	Rare	MDS	Congenital anomalies, B cell deficiency
BLM	Bloom syndrome	Rare	Leukemia, lymphoma	Short stature, sun sensitivity, immune dysfunction
Fanconi anemia[a]	Bone marrow failure syndrome	1 in 130,000	AML, MDS	Multiple congenital anomalies, cancer predisposition, neuroinflammatory syndrome, liver disease, endocrine disorders
Shwachman-Diamond syndrome[b]	Inherited marrow failure	1 in 75,000–150,000	AML, MDS	Pancreatic insufficiency, skeletal abnormalities, liver dysfunction
Dyskeratosis congenita[c]	Telomere biology disorder	1 in 1,000,000	AML, MDS	Skin pigmentation, nail dystrophy, oral leukoplakia, teeth and hair abnormalities, pulmonary fibrosis, immune system disfunction, cancer predisposition
Noonan syndrome[d]	RASopathy	1 in 1000–2500	ALL, JMML	Short stature, cardiac anomalies, facial

				dysmorphism, chest deformities, neck webbing, mild cognitive delays, delayed puberty
CBL syndrome	RASopathy-like disorder	Very rare	JMML	Facial features, developmental delay, cardiac anomalies, hypospadias cutaneous café-au-lait spots (though less frequent than in *NF1*) neurological symptoms.
NFI	Neurofibromatosis type 1 (*NFI*)	1 in 3000	JMML, gliomas	Café-au-lait spots, neurofibromas, Lisch nodules, skeletal abnormalities, neurological and cognitive defects
Down syndrome	Trisomy 21	1 in 700 births	Transitory myeloproliferation, AML	Developmental delay, cardiac anomalies, multiple congenital abnormalities

* Involved genes:

a. *FANCA, FANCB, FANCC, FANCD2, FANCE, FANCF, FANCG, FANCI, FANCJ/BRIP1, FANCL, FANCM, BRCA2/FANCD1, PALB2/FANCN, RAD51C/FANCO, SLX4/FANCP, ERCC4/FANCQ, RFWD3*, and other

b. *SBDS, DNAJC21, EFL1, SRP54*

c. *DKC1, TERC, TERT, TINF2, NOP10, NHP2, RTEL1, ACD, PARN, STN1, WRAP53, ZCCHC8*

d. *PTPN11, SOS1, RAF1, KRAS, NRAS, BRAF, SHOC2, RIT1, LZTR1, SPRED1*

Abbreviations: AML= acute myeloid leukemia; MDS = myelodysplastic syndrome; CMML = chronic myelomonocytic leukemia; ALL = acute lymphoblastic leukemia; CML = chronic myeloid leukemia; MPN = myeloproliferative neoplasm; MM = multiple myeloma; MIRAGE = acronym for *m*yelodysplasia, *i*nfection, *r*estriction of growth, *a*drenal hypoplasia, *g*enital phenotypes, and *e*nteropathy; BM = bone marrow; JMML = juvenile myelomonocytic leukemia.

References:

111. Baliakas, P.; Tesi, B.; Cammenga, J.; Stray-Pedersen, A.; Jahnukainen, K.; Andersen, M.K.; Agerstam, H.; Creignou, M.; Dybedal, I.; Raaschou-Jensen, K.; et al. How to manage patients with germline DDX41 variants: Recommendations from the Nordic working group on germline predisposition for myeloid neoplasms. *Hemasphere* **2024**, *8*, e145. https://doi.org/10.1002/hem3.145.

133 Zhang, J.; Walsh, M.F.; Wu, G.; Edmonson, M.N.; Gruber, T.A.; Easton, J.; Hedges, D.; Ma, X.; Zhou, X.; Yergeau, D.A.; et al. Germline Mutations in Predisposition Genes in Pediatric Cancer. *N. Engl. J. Med.* **2015**, *373*, 2336–2346. https://doi.org/10.1056/NEJMoa1508054.

134 Schwartz, J.R.; Ma, J.; Lamprecht, T.; Walsh, M.; Wang, S.; Bryant, V.; Song, G.; Wu, G.; Easton, J.; Kesserwan, C.; et al. The genomic landscape of pediatric myelodysplastic syndromes. *Nat. Commun.* **2017**, *8*, 1557. https://doi.org/10.1038/s41467-017-01590-5.

135 Feurstein, S.; Churpek, J.E.; Walsh, T.; Keel, S.; Hakkarainen, M.; Schroeder, T.; Germing, U.; Geyh, S.; Heuser, M.; Thol, F.; et al. Germline variants drive myelodysplastic syndrome in young adults. *Leukemia* **2021**, *35*, 2439–2444. https://doi.org/10.1038/s41375-021-01137-0.

136 Sebert, M.; Passet, M.; Raimbault, A.; Rahme, R.; Raffoux, E.; Sicre de Fontbrune, F.; Cerrano, M.; Quentin, S.; Vasquez, N.; Da Costa, M.; et al. Germline DDX41 mutations define a significant entity within adult MDS/AML patients. *Blood* **2019**, *134*, 1441–1444. https://doi.org/10.1182/blood.2019000909.

181 Dokal, I.; Tummala, H.; Vulliamy, T. Inherited bone marrow failure in the pediatric patient. *Blood* **2022**, *140*, 556-570. https://doi.org/10.1182/blood.2020006481.
